# Parental Willingness for COVID-19 Vaccination among Children Aged 5 to 11 Years in Riyadh City, Saudi Arabia: A Cross-Sectional Study

**DOI:** 10.3390/vaccines10121979

**Published:** 2022-11-22

**Authors:** Awad Mohammed Al-Qahtani, Basheerahmed Abdulaziz Mannasaheb, Mohammed Ashique K. Shaikh, Sarah Abdulrahman Alajlan, Mohammed Saeed Z. Alayed, Ibrahim Ahmed Shaikh, Syed Mohammed Basheeruddin Asdaq, Faisal Saeed Al-Qahtani, Eisa Yazeed Ghazwani, Nasser Saeed Al-Qahtani, Bayan Fuad Abbag

**Affiliations:** 1 Department of Family and Community Medicine, College of Medicine, Najran University, Najran 66462, Saudi Arabia; 2 Department of Pharmacy Practice, College of Pharmacy, AlMaarefa University, Dariyah, Riyadh 13713, Saudi Arabia; 3 Pharmacy Services Division, Najran University Hospital, Najran 66251, Saudi Arabia; 4 Department of Pediatrics, College of Medicine, Najran University, Najran 66462, Saudi Arabia; 5 Department of Pharmacology, College of Pharmacy, Najran University, Najran 66462, Saudi Arabia; 6 Department of Family and Community Medicine, College of Medicine, King Khalid University, Abha 61421, Saudi Arabia

**Keywords:** COVID-19 vaccine, vaccine hesitancy, children, Saudi Arabia, parental attitude

## Abstract

To manage the COVID-19 outbreak, the WHO recommends adult and child vaccination. Vaccine skepticism has been a major worldwide health concern for decades, and the situation is worsening. The primary purpose of this study was to investigate parental willingness to vaccinate their children (aged 5 to 11 years) against COVID-19 and to describe its relationship with attitude, barriers, facilitators, and sources of knowledge regarding the vaccine. Methods: From February to March 2022, a community-based cross-sectional survey was undertaken among the parents of Riyadh city, Saudi Arabia. We employed a convenient sampling procedure to gather the required sample. Using the Raosoft sample size calculator, a minimum sample size of 385 was determined based on a 95% confidence level, a 5% margin of error, and a 5% precision level. The data were analyzed using version 26 of SPSS. A *p*-value less than 0.05 was judged statistically significant. The Chi-square test and likelihood ratio were utilized to describe the relationship between socio-demographic characteristics, driving factors, and COVID-19 vaccine hesitancy. Vaccine hesitancy associated factors were identified using multivariate binary logistic regression. A total of 528 replies were received. The majority of respondents were mothers (77.7%), aged 26 to 40 years (67.8%), married (91.5%), Saudi nationals (96.2%), college graduates (70.6%), with a monthly family income of more than SAR 10,000 (46.4%), non-healthcare professionals (84.7%), employed in the government sector (33.7%), with three children (23.3%), and children aged 5 to 11 years (88.7%). A little more than half of the parents (55.7%) exhibited considerable vaccination hesitancy. About 16.28% of parents were willing to vaccinate their children as soon as possible, compared to 38.44% who had no interest whatsoever in vaccination. A greater proportion of mothers and unemployed parents were unwilling to vaccinate their children. Parents with a higher monthly income (above SAR 10,000), who worked as healthcare professionals, and whose children suffered from chronic conditions were significantly more ready to vaccinate their children against COVID-19. Parents who were aware of anti-vaccination campaigns and who vaccinated their children with required childhood vaccines were also much more likely to vaccinate their children against COVID-19. Most parents (66.9%) obtained information on COVID-19 via the Saudi Ministry of Health website, followed by social media (48.1%). The vaccine’s novelty and the dearth of reliable information about its safety (65%) and insufficient information about its effectiveness (36.2%) were the primary reasons for not vaccinating children against COVID-19, whereas preventing children from contracting COVID-19 (55.9%) and government mandate (38.8%) were the primary reasons for vaccinating children against COVID-19. Conclusions: There was significant parental hesitancy to immunize their children against COVID-19. To involve and educate parents, multi-component interventions must be developed and implemented.

## 1. Introduction

As of September 2022, about 603.7 million confirmed cases and more than 6.48 million deaths have been caused by the coronavirus (COVID-19) pandemic worldwide. Most of the confirmed cases (249 million) were from the Europe region [1]. According to the World Health Organization (WHO), the most effective way to control the COVID-19 pandemic is vaccination [2]. The COVID-19 vaccination is being promoted as the most promising intervention for adults and children. Currently, 12,540 million doses of the vaccine have been administered [1]. For many years, hesitancy toward vaccines has been considered a potential global health challenge [3] and regarded as one of the top 10 health threats by the WHO in 2019; hence, it needs considerable attentiveness [4]. The recent development of COVID-19 vaccines has further pushed vaccine hesitancy to the forefront of discussion. Few studies have observed the high prevalence of COVID-19 vaccine hesitancy, making it a potential public health challenge for the healthcare system [5,6,7]. Overall, immunizations continue to play a crucial role in defending susceptible persons against potentially fatal infections that are vaccine preventable. Furthermore, the scientific community agrees that a COVID-19 vaccine can effectively prevent the extremely contagious COVID-19, which the WHO proclaimed a pandemic in March 2020 [8]. Vaccine hesitancy is the practice of delaying or forgoing vaccination despite the availability of vaccination services. This practice may lower vaccination rates, which in turn raises the risk of infectious disease outbreaks and epidemics. A few variables, such as concerns with confidence (do not trust a vaccination or a physician), complacency (do not recognize a need for a vaccine or do not value the vaccine), and convenience, are described as contributing factors for vaccine hesitation [9]. 

COVID-19, which is brought on by the SARS-COV-2 coronavirus, exhibits a range of respiratory tract symptoms, including fever, shortness of breath, pneumonia, and disease that resembles the flu [10,11]. In Saudi Arabia, COVID-19 has resulted in 813,986 confirmed cases and 9309 deaths as of September 2022 [12]. New SARS-COV-2 variants with different features have started to appear because of mutations in the virus genome. Furthermore, these variants may increase the disease infectivity and severity and challenge the efficacy of the vaccine [13]. As a result, the ongoing COVID-19 public health crisis has had a devastating effect on every element of human existence, leading to notable morbidity and mortality, unfavorable psychological effects, and increasing socioeconomic losses. The epidemic has also derailed the toilsome progress made in the previous 10 years to raise vaccination rates. In Saudi Arabia, COVID-19 vaccination was made mandatory for adults, without which they could not resume their job, travel abroad, or visit healthcare facilities and shopping malls. However, no such conditions were applied to children. In June and December 2021, Saudi Arabia started giving the COVID-19 vaccine to children aged 12–18 years and 5–11 years, respectively [14,15]. The Saudi Food and Drug Authority approved the use of the Pfizer-BioNTech COVID-19 vaccine for children between the ages of 5 and 11 years [16]. In the last decade, there has been an increase in the rate of parents refusing or delaying vaccines for their children, and low rates of vaccination have contributed to the resurgence of many vaccines preventable diseases.

Factors responsible for low vaccine uptake include access issues such as vaccine availability, convenience, cost, and motivation. The term “motivation” refers to the overlapping constructs of intention, willingness, acceptability, and hesitancy toward vaccinations (e.g., perceived risk of disease, faith in vaccine effectiveness, safety concerns related to vaccinations and vaccination administration), as well as the social environment (e.g., the strength of provider recommendation, social norms surrounding vaccinations, myths and misinformation about vaccinations) [17].

There has been considerable vaccine disinformation propagated throughout the pandemic, and there have been worries that vaccine hesitancy has increased; the other potential factors responsible for vaccine hesitancy are a low literacy rate, socioeconomic disparities, cultural and religious beliefs, and belief in conspiracy theories (such as the idea that vaccines might harm or sterilize children or contain substances derived from monkeys or pigs) [18].

The parents are usually decision makers for their children who are aged below 11 years; therefore, it is essential to understand the parents’ willingness, attitude, source of information about the COVID-19 vaccine, and related facilitators and barriers. The primary objective of the present study was to explore the parents’ willingness to vaccinate their children (aged 5–11 years) against COVID-19 and to describe its relationship with attitude, barriers, facilitators, and sources of information about the vaccine. This is the first study conducted exclusively in Riyadh city, Saudi Arabia, to explore parental COVID-19 vaccine hesitancy.

## 2. Methodology

### 2.1. Study Setting and Design

The present community-based cross-sectional study was conducted among the parents of Riyadh city, Saudi Arabia, to explore their attitude and hesitancy to vaccinate their children against COVID-19. The study duration was from February to March 2022. The study was approved by the Institutional review board (IRB) of AlMaarefa University with registration number-IRB06-21022022-17, dated 16 February 2022. The online questionnaires were distributed as Google forms. The study purpose and informed consent were displayed at the start of the online form, and parents were allowed to either accept or reject their participation, making participation voluntary. Parents who clicked decline were directed to the finish participation page, and their responses were not recorded.

### 2.2. Sampling Technique, Sample Size and Distribution of Study Tool

We used a convenient sampling technique to collect the required sample. As of 2022, the total population of Riyadh city is estimated to be 7,538,200 as reported by the world population review (https://worldpopulationreview.com/world-cities/riyadh-population) [19]. Assuming the 95% confidence level, a margin of error of 5% and a sample proportion of 50%, a minimum sample size of 385 was calculated using the Raosoft sample size calculator (http://www.raosoft.com/samplesize.html, accessed on 5 January 2022). The barcode of the study tool was displayed at the entrances of public places such as shopping malls, indoor kids playing areas, supermarkets, and children’s parks. Additionally, we shared the survey link through social platforms such as Twitter, LinkedIn, Facebook, and WhatsApp status.

### 2.3. Development of Study Tool and Validation

A substantial review of the relevant literature was done [5,6,7,20,21,22,23,24,25,26] to draft the initial study tool, followed by validation from experts in the field of microbiology, epidemiology, pediatrics, and community health. The questionnaire was modified as per the suggestions from the experts. The questionnaire was translated into an Arabic version through an independent professional translator. A pilot study was carried out by recruiting 50 parents (25 fathers and 25 mothers) to confirm the face validity. A few questions were incorporated at the end of the survey to gather the parents’ feedback about the study tool including “Are questions written in easy and understandable language” and “Are any questions not clear or difficult to understand”. Necessary changes were made considering the parents’ feedback. The reliability of the study tool was confirmed by calculating the Alpha Cronbach factor (0.75). Finally, a bilingual (Arabic and English) online questionnaire was disseminated to the study participants. Participants could fill the survey only once using their email account. Repeat responses were only possible if participants could fill it using a different device and/or using another email account, which is extremely rare.

### 2.4. Sections of the Study Tool

#### 2.4.1. Demographic Details

The first section recorded sociodemographic details about the parents and their children, including parents’ age, nationality, marital status, education, monthly income, job section, relationship with the child, number, age and gender of children, and presence of any chronic disease among children. No certain age range was fixed to participate in the study. However, after collecting all responses, we categorized the age domain into 3 suitable categories of 25 years and below, 26 to 40 years, and 41 years.

#### 2.4.2. Parental Behavior about COVID-19 and Immunization History of Child

This section documented the parents’ commitment to following the precautionary measures against COVID-19, did they see or heard about any anti-COVID-19 vaccine campaign, COVID-19 infection of any direct family member and severity of symptoms, COVID-19 vaccination status of parents, and post-vaccination adverse symptoms. Additionally, this section collected the information about immunization history of children regarding mandatory childhood vaccination and post-vaccine symptoms. These behaviors were considered to be potential factors which influence the parents’ intention to vaccinate their children against COVID-19.

#### 2.4.3. Measuring Vaccine Hesitancy-Parents’ Willingness to Vaccinate Their Children against COVID-19

This section documented the parents’ willingness and intention to vaccinate their children against COVID-19 using a Likert scale option, including not willing to vaccinate, undecided, delay for a few months or a year, and vaccinate as soon as possible [20,23]. Parents were hesitant if they selected unwilling, undecided, or delayed vaccinating options, whereas vaccine acceptance was considered among parents who intended to vaccinate their children as soon as possible.

#### 2.4.4. Parental Attitude and Vaccine Hesitancy Score (VHS) towards COVID-19 Vaccination in Children

This domain of questionnaire measured the parents’ attitude regarding importance of COVID-19 vaccination among children using the tool created by the Strategic Advisory Group of Experts (SAGE) on Immunization of the WHO [20]. As per Temsah et al., 2021, the statement “All childhood vaccines offered by the government program in my community are beneficial” was eliminated from the tool to boost its application and better suit the study group. We used the nine statements addressing the importance of vaccination in children, in which six statements evaluated the positive attitude, whereas three statements documented the parents’ concerns regarding vaccine and vaccination [21]. We further modified the tool by replacing the statement “I am concerned about serious adverse effects of COVID-19 vaccine” with “I think the producers of the COVID-19 vaccine are concerned about my child’s health?” In line with Kempe et al., 2020, we used a 4-point Likert scale [22]. Because current research indicates that a 4-point scale lacking a neutral option reduces the likelihood of social conformity, the option “neutral” was removed.

The mean parental attitude (VHS) score toward vaccines was calculated by averaging the nine items on the scale [21] after reverse ordering the negatively worded statements. A higher score indicated a better attitude toward vaccination, and then VHS scores were categorized into low vs. high hesitancy toward vaccines based on a cut off value (=3 points); thus, people with a mean attitude score below 3 were considered to have high hesitance, whereas a score of 3 and above indicates low parental hesitance toward COVID-19 vaccination among children. There are 9 statements used to measure parental attitude. Each statement has 4 options; strongly agree, agree, disagree, and strongly disagree. Thus, there is scoring of 4, 3, 2, and 1 for positive statements and reverse ordering for negatively worded statements. We took the average score of nine statements. If the average was 3 and above, it was considered low hesitancy and an average score of less than 3 was considered high hesitancy. These cut off points were adapted from a previously published study [21].

#### 2.4.5. Source of Information and Reasons Influencing Parents’ Intention to Vaccinate Their Children

This section collected information from sources referred by the parents to acquire information about COVID-19 vaccination among children. The participants were allowed to select multiple sources of information, including the Saudi ministry of health website, social media, television, WHO, physicians, friends, YouTube videos, etc. Parents also mentioned the most common reasons which either encourage or discourage them from vaccinating their children against COVID-19. Multiple answers were allowed in this section. The reasons for vaccinating children were “to prevent disease occurrence”, “compulsion from the government”, “to get my child into a day-care center or school”, “due to travel restrictions”, and “pressure from family and friends”. The reasons for not vaccinating children were “lack of adequate data about the safety of new vaccine”, “insufficient information about the effectiveness of vaccine”, “fear of its adverse effect on children health in future”, etc.

### 2.5. Data Processing and Statistical Analysis

The collected responses were checked for their consistency and completeness. Repeat and incomplete responses were not included in the analysis. Data were analyzed using SPSS version 26. A *p*-value of less than 0.05 was considered significant. The Chi-square test and likelihood ratio were used to describe the association between sociodemographic variables, influencers, and vaccine hesitancy towards COVID-19. Factors associated with vaccine hesitancy were determined using multivariate binary logistic regression.

## 3. Results

Altogether we collected 528 responses, and the same were subjected to analysis. Table 1 depicts the demographic details of the study participants. The majority of respondents were mothers (77.7%), aged 26 to 40 years (67.8%), married (91.5%), Saudi nationals (96.2%), holding college degrees (70.6%), with a monthly family income of more than SAR 10,000 (46.4%), non-healthcare professionals (84.7%), working in government sector (33.7%), with three children (23.3%), both boys and girls (56.6%), aged 5–11 years old (88.4%), with no chronic diseases (78.6%).

### 3.1. Parents’ Willingness to Vaccinate Their Children against COVID-19

The association between sociodemographic details with parents’ willingness to vaccinate their children against COVID-19 is illustrated in Table 2. Our findings indicate a significant impact of parents’ relationship (father/mother), monthly income, working area (healthcare professional), job sector, and presence of chronic disease among children on parents’ willingness to vaccinate their children against COVID-19. A significantly (*p* = 0.004) higher number of mothers (41.7%) have shown denial to vaccinate their children compared to fathers (27.1%). Vice-versa, a higher number of fathers (18.6%) were willing to vaccinate their children compared to mothers (15.6%). Likewise, parents with a family’s monthly income of more than SAR 10,000 were significantly (*p* = 0.023) highly (20.4%) willing to vaccinate their children as soon as possible compared to parents with a monthly income of SAR 5000–10000 (10.3%), SAR <5000 (17.9%), and preferred not to disclose their income (13.7%). Similarly, parents working in the healthcare profession (HCP) have shown significantly (*p* < 0.001) higher (28.4%) willingness to vaccinate their children against COVID-19 compared to non-healthcare professional parents (14.1%). Unemployed parents have shown significantly (*p* = 0.040) higher (56.3%) reluctance to vaccinate their children compared to employed parents. Moreover, parents with children suffering from long-term/chronic disease have demonstrated significantly (*p* = 0.018) higher (19.5%) eagerness to vaccinate their children compared to their counterparts (15.4%). We did not notice any significant impact of parents’ age, nationality, marital status, education, or number and gender of children on willingness to vaccinate their children against COVID-19.

### 3.2. Vaccine Hesitancy Scale (VHS) and Attitude Regarding the Importance of Vaccinating Children against COVID-19

The attitude regarding the importance of the COVID-19 vaccine among children was measured using nine statements. Parental COVID-19 vaccine hesitancy was measured using VHS. A score of 3 and above was considered low vaccine hesitancy, whereas a score of less than 3 was considered high hesitancy regarding COVID-19 vaccination among children. In the present findings, we observed that more than half of parents (55.9%) were highly hesitant, and 44.1% had shown low hesitancy toward COVID-19 vaccination for their children.

**Figure 1:**
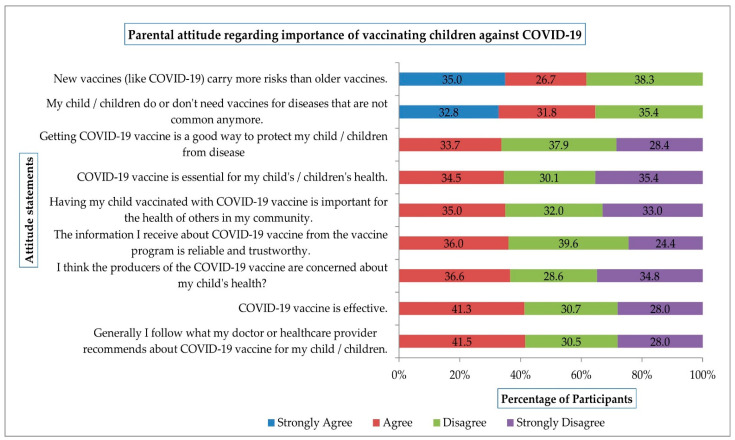
Parental attitude regarding the importance of COVID-19 vaccination among children.

More than 60% of parents showed their agreement with statements, “New vaccines (like COVID-19) carry more risks than older vaccines” and “My child/children do or don’t need vaccines for diseases that are not common anymore”, indicating that parents are concerned about the risk of COVID-19 vaccines among children and consider child vaccination unnecessary as the spread of COVID-19 is under control. Additionally, about one-third of parents agreed (33.7%) that “Getting COVID-19 vaccine is a good way to protect my child/children from disease”, 34.5% of parents agreed that “COVID-19 vaccine is essential for my child’s/children’s health”, 35% believed that “Having my child vaccinated with COVID-19 vaccine is important for the health of others in my community”, 36% of parents agreed that “The information I receive about COVID-19 vaccine from the vaccine program is reliable and trustworthy”, and 36.6% of parents declared that “I think the producers of the COVID-19 vaccine are concerned about my child’s health”. More than 40% of parents agreed that the “COVID-19 vaccine is effective” and “Generally I follow what my doctor or healthcare provider recommends about COVID-19 vaccine for my child/children” (Figure 1).

### 3.3. Parental Behavior towards COVID-19 Vaccination, Precautionary Measures, and Childhood Vaccination

More than half of parents (62.1%) have seen or heard of anti-vaccine campaigns. Most parents (36.2%) showed little commitment to following precautionary measures against COVID-19; however, nearly one-fourth (22.3%) of parents described their great commitment to following precautionary measures. More than three-fourths (75.6%) of parents mentioned having a direct family member infected with COVID-19 with mild (24.1%) to moderate (39.1%) symptoms. However, 2% of parents indicated having a direct family member who died due to COVID-19 infection. Altogether, 92.4% of parents have received COVID-19 vaccination shots, with mild (31.6%) to moderate (54.2%) post-vaccine symptoms. About 77.8% of parents stated vaccination of their children with mandatory childhood vaccines with mild (42.8%) to moderate (47.2%) post-vaccine adverse symptoms (Table 3).

### 3.4. Parental Source of Information about COVID-19 Vaccine

**Figure 2:**
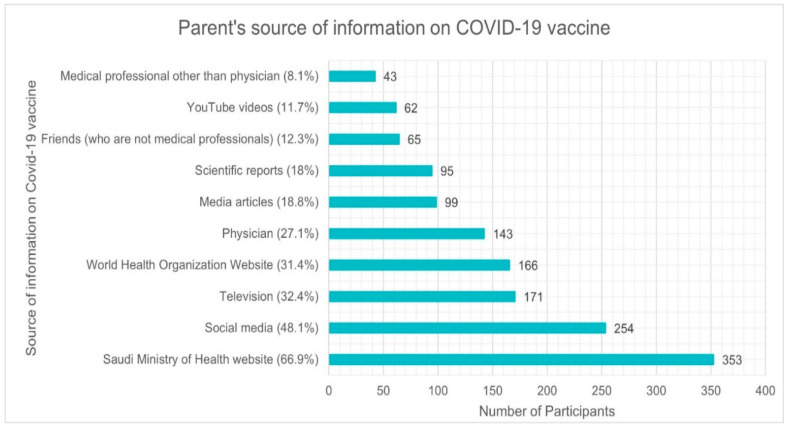
Parents’ source of information on COVID-19 vaccine in children.

Figure 2 depicts the numerous sources used by parents to acquire information about COVID-19 vaccination among children. Overall, 66.9% of parents referred to the Saudi Ministry of Health website to obtain information about the COVID-19 vaccine among children, followed by social media (48.1%), television (32.4%), the WHO (31.4%), physicians (27.1%), and media articles (18.8%). Only a few parents used YouTube videos (11.7%), non-medical professional friends (12.3%), and medical professionals other than physicians (8.1%). About 14.9% of parents mentioned contacting friends to obtain COVID-19 related information.

### 3.5. Reasons Influencing the Parent’s Decision to Vaccinate Their Children against COVID-19

**Figure 3:**
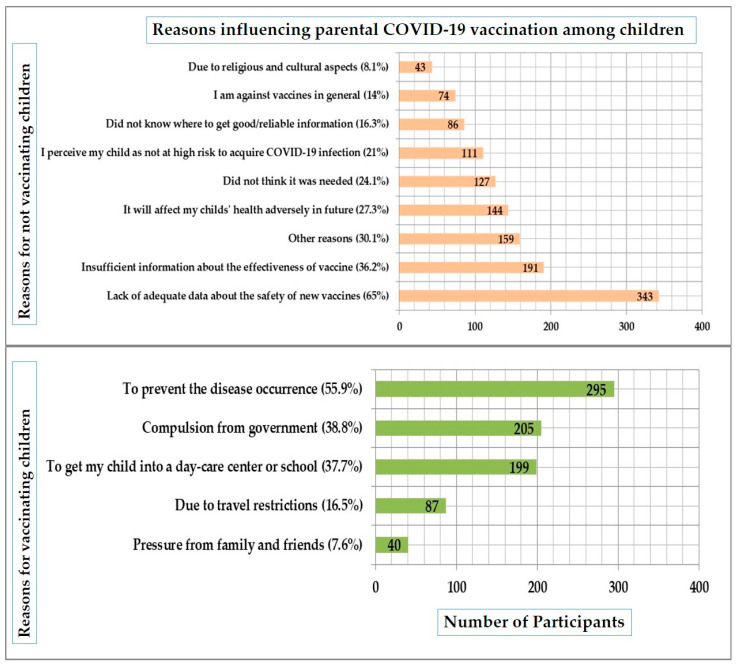
Reasons influencing parents’ decision to vaccinate their children against COVID-19.

Figure 3 illustrates the reasons impacting the parents’ decision to vaccinate their children against COVID-19. The most common encouraging reason mentioned by parents includes prevention of disease occurrence among their children (55.9%) followed by obligation from the government (38.8%) and to enter their children into day care or school (37.7%). About 16.5% and 7.6% of parents mentioned travel restrictions and pressure from family and friends, respectively, as a reason for vaccinating their children. Conversely, the most common reason for not vaccinating children against the COVID-19 vaccine was a lack of adequate data about the safety of the new vaccine (65%), followed by insufficient information about the effectiveness of the vaccine (36.2%), fear about the adverse impact of the COVID-19 vaccine on the child’s health in future (27.3%), considering this vaccine as non-essential among children (24.1%), and considering their child at low risk of getting infected with COVID-19 (21%). However, a few parents mentioned religious and cultural aspects (8.1%) and anti-vaccine beliefs (14%) as discouraging reasons to vaccinate their children against COVID-19 (Figure 3).

### 3.6. Drivers of Parental COVID-19 Vaccine Hesitancy for Their Children

Table 4 displays the specific drivers influencing the parent’s willingness to vaccinate their children against COVID-19. We performed a chi-square analysis to predict the significant impact of potential contributors. Results indicate a significant impact of the anti-COVID-19 vaccination campaign (*p* = 0.011) and earlier mandatory childhood vaccination (*p* < 0.001) on parents’ willingness to vaccinate their children against COVID-19. The parents who have heard of the anti-vaccine campaign had a significantly high (20.4%) willingness to vaccinate their children against COVID-19 as soon as possible compared to parents who have not heard of the anti-vaccine campaign (9.5%). Conversely, parents who have vaccinated their children with mandatory childhood vaccination showed a significantly high (19.5%) willingness to vaccinate their children against COVID-19 as soon as possible compared to parents who did not vaccinate their children with childhood vaccines (5.1%). We did not observe any significant impact of the family’s commitment towards precautionary measures, parents’ COVID-19 vaccination status, and COVID-19 infection of any direct family members.

### 3.7. Binary Logistic Regression Analysis to Identify the Factors Associated with Parental COVID-19 Vaccine Hesitancy

The variables showing a significant influence on parental vaccine hesitancy in the chi-square test (parents’ relationship, family’s monthly income, working area, job sector, presence of chronic disease among children, anti-vaccine campaign, and childhood vaccine history) were subjected to binary logistic regression analysis to determine the individual factor of parental vaccine hesitancy. The variables such as parents working as HCP (B: −0.697, *p* = 0.040), anti-vaccine campaign (B: −0.993, *p* = 0.001), and childhood immunization (B: −1.614, *p* < 0.001) showed the significant negative impact on parents’ willingness to vaccinate their children against COVID-19, indicating that parents who work as HCP, have heard/seen about the anti-vaccine campaign, and completed mandatory childhood vaccination are more likely to vaccinate their children compared to their counterparts. Parents working as HCP are 0.5 times less likely to be hesitant compared to parents working as non-HCP. Similarly, parents who have heard/seen anti-vaccine campaigns are 0.37 times less likely to be hesitant compared to their counterparts. Likewise, parents who did mandatory childhood vaccination for their children are 0.2 times less likely to be hesitant. All other variables were non-significantly associated with parents’ vaccine hesitancy toward COVID-19 (Table 5).

## 4. Discussion

The study’s results shed a great deal of light on the context of vaccine skepticism among the parents in Riyadh city. A correlation between parents’ socioeconomic status and their openness to vaccinating their children against COVID-19 was found in the study. Concerns and misunderstandings about the significance of safeguarding children from COVID-19 are addressed, as well as parents’ perspectives on the significance of vaccinating children against the virus and their behavior in response to vaccination, preventative measures, and mandated childhood vaccinations. These results are a valuable addition to the existing literature on parental vaccine hesitance and will help in the design of programs to reduce this issue.

Vaccinations, which are considered one of the most successful therapies ever developed, have helped to avoid and controlled infectious disease epidemics, assisted people in living longer, healthier lives, and saved millions of lives across the world [27]. Vaccination is the best way to achieve herd immunity, which is extremely important. According to a recent study, the population threshold for developing COVID-19 herd immunity spans anywhere from 55 to 82 percent, depending on the biological, environmental, and socio-behavioral components involved [28]. Nonetheless, in order to attain this goal, vast populations, including children, will need to be immunized. In addition, it is crucial to the success of any vaccination program for individuals to be open to the idea of using vaccines not only for themselves but also for their children.

It is known that a variety of factors, including sociodemographic features, cultural and religious viewpoints, political beliefs, and economic beliefs, influence parents’ willingness to vaccinate their children across time, between countries, and even within a single region. Thus, it becomes crucial to study these factors.

In our study, significantly more mothers than fathers were opposed to vaccinating their children against COVID-19, comparable to findings from other studies conducted in Saudi Arabia and Japan. In a national poll of Saudi parents of 5- to 11-year-old children, mothers were more hesitant than fathers to vaccinate their children against COVID-19 [29]. Being a mother was also associated with an increased likelihood of parental COVID-19 vaccine hesitation in Japan [30]. In contrast to the results of our study, mothers with a positive attitude in Poland were eager to vaccinate their children as soon as feasible [23]. In contrast, several studies conducted in Saudi Arabia found no correlation between parental gender and willingness to vaccinate their children against COVID-19 [21,31].

Numerous studies have demonstrated that monthly household income influences parental vaccine hesitancy and their willingness to vaccinate their children against COVID-19. Similar to the findings of other studies, parents in our study with a monthly household income of more than SAR 10,000 were more inclined to vaccinate their children with COVID-19 vaccines. In research conducted among parents of children aged 5 to 11 in Najran city, Saudi Arabia, those with a monthly household income of more than SAR 10,000 were much more likely to vaccinate their children against COVID-19 [31]. In Illinois, U.S.A., parents with an annual household income of more than USD 150,000 exhibited less COVID-19 vaccine hesitation than parents with an annual household income of fewer than USD 40,000 [24]. In England, participants with a lower household income were 1.8 times more likely to refuse a COVID-19 vaccination for their children [5]. In Bangladesh, parents of children from households with a lower income exhibited significantly greater COVID-19 vaccine hesitancy [32].

In our study, parents’ employment status influenced their willingness to vaccinate their children against COVID-19, with unemployed parents being more hesitant. In a study conducted in Bangladesh, it was discovered that unemployed parents exhibited significantly greater COVID-19 vaccine hesitancy [32]. This could be explained by the terrible impact the COVID-19 epidemic had on global economies, resulting in the loss of full or partial employment and leaving families with insufficient or no income to meet their fundamental necessities and sustain them. Similar to the findings of Temsah et al., 2021 [21], and Aedh, 2022 [31], parents in our study who worked as HCPs showed significantly higher willingness to vaccinate their children against COVID-19. The fact that healthcare professionals are at the forefront of vaccination programs and are aware of the significance of vaccinating against COVID-19 in achieving herd immunity and preventing its further spread may contribute to a greater acceptance of COVID-19 vaccination among parents for their children.

In contrast to the results reported in Najran, one of the good outcomes of our study is that parents of children with chronic diseases were substantially more inclined to vaccinate their children. In a study conducted in Najran, it was discovered that parents of children with chronic diseases were 9.9 times less likely to vaccinate their children against COVID-19 [31]. It is to be noted that, at the onset of global vaccination, several health authorities and organizations prioritized the immunization of high-risk persons, including those with chronic conditions.

Several studies have demonstrated that parents of children who have received all required childhood vaccines had less vaccine hesitancy and a greater willingness to vaccinate their children against COVID-19. In our study, parents who immunized their children with mandatory childhood vaccines were significantly more likely to vaccinate them with the COVID-19 vaccine. Other researchers have discovered similar results. In the Saudi Arabian city of Najran, childhood vaccination was found to have a beneficial effect on COVID-19 vaccination, with parents of children who had gotten the required childhood immunization being 0.4% more likely to vaccinate their children against COVID-19 [31]. In a national poll conducted in Saudi Arabia, parents of 5- to 11-year-old children who had gotten an annual influenza vaccination were less hesitant to vaccinate than parents of children who had not had an annual influenza vaccination [29]. In Bangladesh, parents who did not or would not vaccinate their children with routine immunizations exhibited significant hesitation towards the COVID-19 vaccine [32]. The conclusion is that parents who vaccinate their children with required childhood immunizations are aware of and comprehend the significance of vaccination.

Anti-vaccination movements in certain places of the world, such as the Middle East, are an additional element that contributes to vaccine hesitancy. Anti-vaccination campaigns that promote false, twisted, deceptive, and misleading information about infectious diseases and vaccines also pose a major risk to the health of children. It is particularly important to monitor social media because it is a breeding ground for the anti-vaccine sentiment. So that more people can be inoculated against COVID-19 and protected, anti-vaccination groups must be continuously watched and actively controlled [33,34,35]. Despite their awareness of anti-COVID-19 vaccination programs, parents were much more ready to vaccinate their children with the COVID-19 vaccine than parents who were oblivious of anti-COVID-19 vaccination campaigns. This is associated with parental comprehension of the COVID-19 vaccination and its relevance. In addition, the study was conducted in Riyadh, one of the major cities and the capital of Saudi Arabia, which has one of the highest human development indices, a highly literate population, a number of universities and educational institutions, the ministry of health, and an abundance of advanced healthcare facilities, etc., to meet the needs of its population. These reasons may have influenced parental vaccination attitudes against COVID-19.

In our study, 55.9% of parents scored less than 3 on the Vaccine Hesitancy Scale, indicating that they are highly vaccine-hesitant. About 203 parents (38.44%) would not vaccinate their children against COVID-19, whereas just 86 parents (16.28%) would do so immediately. In a study conducted by Temsah et al., 2021, 45% of surveyed parents were reluctant to vaccinate their children against COVID-19, with 50% of these parents not intending to vaccinate and 22.3% deciding to vaccinate [21]. According to another national study, 61.9% of parents of children aged 5 to 11 years were hesitant to vaccinate their children [29]. In a study conducted in the city of Najran, 72.2% of polled parents displayed high hesitancy about vaccinating their children against COVID-19 [31]. According to another cross-sectional survey, 56% of Saudi Arabian parents were not willing to vaccinate their children against COVID-19 [26]. In Illinois, 33% of parents were hesitant to get their children vaccinated against COVID-19 [24]. In research from Vietnam and China, parents of children aged 5 to 17 years and 3 to 4 years were found to have lower vaccine hesitancy rates of 26% and 20.7%, respectively [36,37]. According to a survey performed in Poland, 44.1% of parents are ready to vaccinate their children as soon as possible, while 25.8% are completely opposed [23]. More than 85 percent of mothers and mothers-to-be in Brazil, Colombia, India, and Mexico stated they would be willing to have their children immunized against COVID-19, according to a survey conducted in 16 countries [25]. In addition, in China, 89% of parents expressed willingness or extreme willingness to vaccinate their children against COVID-19 [37].

To further examine the elements that may impact vaccine hesitancy among parents and their decision to vaccinate their children against COVID-19, it is necessary to identify and study the sources from which parents obtain information about COVID-19 and vaccines.

The Saudi MOH website was the most often cited source of information by parents (66.9%), similar to the findings in Najran city (75.9%) [31]. In one of the Saudi national surveys, 85.9% of parents accessed the MOH website and were significantly more likely to vaccinate their children against COVID-19 [21]. In another Saudi nationwide poll of parents of children aged 5 to 11, 37.3% of parents cited MOH resources as a source of information regarding COVID-19 [29]. In this study, 31.4% of polled parents visited the WHO website, which is slightly higher than the proportion of parents in Najran city (25.9%) [31]. In research done by Temsah et al., 2021, however, a higher proportion of parents (43.4%) had visited the WHO website and significantly less anticipated vaccinating their children with the COVID-19 vaccine [21]. About 52% of parents in Illinois, United States, referred to government entities as a source of information, and 48% expressed confidence in the source; this was associated with a decreased likelihood of vaccine hesitancy [24]. The most reliable source of information for COVID-19 in Japan was also government/public organizations (26.7%) [30], and in China, parents who worked in public institutions had a lower vaccine hesitancy rate [37]. As routine childhood vaccinations and now COVID-19 vaccinations are administered almost exclusively by the public healthcare system in Saudi Arabia, increasing parental trust in the healthcare system and government agencies can significantly increase their willingness to vaccinate their children against COVID-19.

Due to their education and specialized training, HCPs, particularly family physicians, pediatricians, and members of vaccination teams, can play a vital role in educating and persuading parents to make favorable decisions regarding the immunization of their children against COVID-19. In our study, a greater proportion of parents (27.1%) received information about the COVID-19 vaccine from physicians than from parents in Najran city (15.3%) [31], while a smaller proportion of parents (8.1%) received information from medical professionals other than physicians than the 17.5% of parents in Najran city [31]. In the US, 40% of the parents who were studied got their information from HCPs, and 44% of them had faith in them, which was linked to lower odds of vaccine hesitancy [24]. In a Polish study, parents who consulted their physicians were more willing to vaccinate their children immediately [23]. In Italy, pediatricians urging that children be fully immunized had a substantial impact on parental vaccination decisions. Pediatricians and other physicians were the most sought and trusted information sources among both pro- and anti-vaccine parents [37]. Parents can benefit from a targeted and successful communication strategy employed by HCPs. Particularly, HCPs working in primary healthcare centers where COVID-19 immunizations are administered can influence parents’ decisions to vaccinate their children.

When compared to the results of the Najran study, where 32.8% and 16.6% of parents used TV and media articles as their source of information, respectively [31], in our study 32.4% and 18.8% of parents used TV and media articles as their source of information, respectively. In a Saudi national survey of parents of children ages 5 to 11, 16.2% of parents said that they learned about COVID-19 from the TV or radio [29]. In Japan, 16.7% of parents used public news sources, and 22.3% of parents used private news sources [30]. Public and private electronic and print media can be utilized to disseminate information and aid in persuading parents to vaccinate their children against COVID-19.

About 48.1% of the people in our study got information from social media, higher than the findings of Temsah et al., 2021 [21] and Almalki et al., 2022 [29], wherein 35.4% and 37.9% of parents had used social media, respectively. However, only 6.9% of Najran city parents [31] and 5% of Japanese parents [30] turned to social media for COVID-19 updates. In line with the findings of Temsah et al., 2021 (14.4%) [21], a similar percentage of parents (11.7%) watched YouTube videos. A smaller proportion of Najran city parents (4.3%) consulted YouTube videos for COVID-19 information [31]. Moreover, three-quarters (78.6%) of Polish [23] and 67.2% of American parents [24], respectively, utilized the internet to learn about COVID-19. Although 67% of US parents used the internet, only 26% of those parents had faith in it as a reliable source of information [24]. About 60% of Italian parents said they use the internet to do research, but only 33% said they trusted it as much as they should [38]. In Japan, the proportion of parents who do not want to vaccinate their child increased by three times among those who rely on social media as their primary information source [30]. When researching COVID-19, it is important to proceed cautiously when using social media or other online resources, as the material provided there is often contradictory and must be evaluated thoroughly. Furthermore, these sites serve as a fertile environment for the dissemination of misinformation and the growth of anti-vaccination groups.

It is equally essential to investigate and analyze any variables or concerns that may promote or discourage parents from vaccinating their children against COVID-19.

Similar to the findings of the Najran study, in which 51.3% of parents mentioned this as the primary reason for not vaccinating their children against COVID-19 [31], the lack of adequate data regarding the safety of the new vaccine was cited as the primary reason in our survey by 65% of parents. In a survey conducted by Temsah et al., 2021, 69% of parents mentioned this issue as a concern [21]. In the study by Almalki et al., 2022, 48.3% of parents of 5 to 11-year-old children who initially declared that they would not vaccinate their children indicated that they would register their children to receive the vaccination if they received sufficient information about it [29]. In research by Temsah et al., 2021, 60.6% of parents cited vaccine side effects as a key concern [21], and the same was true in England, where 62.1% of participants cited safety concerns as one of the primary hurdles [5]. Concerns about side effects (81.6%) and vaccine safety (76.3%) were identified as the most prevalent reasons for parental COVID-19 vaccine hesitancy in Vietnam [36]. In Japan, 64% of parents questioned the safety of vaccines or feared their side effects [30]. In Romania, 47.32% said that COVID-19 vaccines were too new and should be examined more, while 24.42% were concerned about COVID-19 vaccine side effects [39]. In the city of Najran, 17.5% of parents cited adverse vaccine reactions as a reason for not vaccinating their children [31]. About 56% of parents in Poland said that the vaccination had not been thoroughly tested [23]. In our study, 27.3% of parents were also concerned about the potential delayed adverse consequences of vaccines on their children’s health. However, in the city of Najran, a greater proportion of parents (43.1%) were concerned that their children may have future complications as a result of the COVID-19 vaccine [31]. Similarly, in Poland, 51.3% of parents stated concerns regarding complications that may arise in the future [23].

Insufficient information regarding the vaccine’s efficacy was stated by 36.2% of parents as a reason for not vaccinating their children against COVID-19. In Poland, 23.5% of parents cited ineffectiveness as a reason for not vaccinating their children, and more than 50% of these parents would never vaccinate their children [23]. About 22% of parents in the Saudi Arabian city of Najran were similarly concerned about the effectiveness of COVID-19 vaccines [31]. In Bangladesh, parents who lacked faith in the efficacy of the COVID-19 vaccination for their children exhibited significant vaccine hesitancy [32]. Safety and efficacy concerns were revealed to be important predictors of parental vaccine hesitancy in a study by Almalki et al., 2022 [29].

Moreover, another reason for not vaccinating the children was the perception that the child was not at high risk of contracting COVID-19 (21%), similar to the findings of Aedh, 2022 [31] and Temsah et al., 2021 [21] in which 19% and 26.1% of parents perceived their child to be at low risk of contracting COVID-19 infection, respectively. About 19% and 26.6% of parents in these studies judged their children to be at low risk for developing complications if they contracted COVID-19 [21,31]. Similarly, 19% of parents in England said that COVID-19 rarely affects children [5].

In our study, 16.3% of parents did not know where to acquire credible information on COVID-19 vaccination for children. A minority of polled parents in England identified a lack of vaccine information as a factor for their refusal to vaccinate their children. Parents also expressed a desire for information regarding the development, efficacy, and safety of vaccines [5]. It is essential to provide parents with accurate information regarding COVID-19 vaccination in order to boost vaccine acceptability for their children. In addition, approximately 14% of parents in the current study were anti-vaccine in general, similar to data from Japan, where 15% of parents were anti-vaccine in general [30]. In the study done by Temsah et al., 2021 [21], and in Romania [39], 21.4% and 22.13% of parents, respectively, disagreed with vaccination in general. In Najran, 10.1% of questioned parents were opposed to vaccination in generally [31].

Nevertheless, there were other factors that led parents to vaccinate their children against COVID-19. Around 56% of parents claimed disease prevention as a reason for vaccinating their children against COVID-19, which was higher than in England [5], where 42% of parents cited this as a reason for vaccinating their children. Compulsion from the government and the need to enroll a child in daycare or school was mentioned by 38.8% and 37.7% of parents, respectively. Students aged 12 and above in Saudi Arabia were required to receive two doses of a COVID-19 vaccination before the start of the academic year in 2021 [40]. This could be a motivator for parents to vaccinate their children against COVID-19. About 16.5% of parents cited travel restrictions.

To be noted, more than 90% of 12 to 18-year-old children were inoculated against COVID-19 by September 2021, and a total of 67,701,901 vaccine doses were administered in Saudi Arabia as of 3 September 2022 [1]. These results hold promise that parents will ultimately vaccinate their children with COVID-19 vaccines, if not immediately, then soon.

Suggestions: Multi-component interventions and unique approaches are needed to counteract vaccine reluctance among parents. Children must have the COVID-19 vaccine. When talking to parents about child vaccines, one should start early and adopt an optimistic attitude. Parents should know the importance of COVID-19 vaccination, and getting the vaccine should be made a mandatory procedure for children. Concerns about vaccine safety, side effects, and effectiveness must be addressed. Parents should know about the rigorous processes utilized during vaccine research and testing on humans, the role worldwide and regional health authorities, including the SFDA, played in analyzing safety and efficacy data, and the continuous post-marketing surveillance. Trusted and reliable channels should be used to disseminate any updated information regarding COVID-19 vaccines in children. HCPs working in vaccination campaigns should be sensitive and share their experiences with parents. Training for healthcare workers should focus on counselling skills and boosting confidence in the public system. These can help build trust with parents and convince them to vaccinate against COVID-19. Furthermore, surveys gauging parental unwillingness to vaccinate their children against COVID-19 should be carried out at various times and in various parts of Saudi Arabia, and future interventions should be shaped in part by the findings of these surveys. Last but not least, it is crucial to take firm action against individuals and groups that spread false and misleading information about COVID-19 vaccines.

Limitations: There are a few limitations to this study. The cross-sectional design shows parents’ replies at one time, and thus it is challenging to track parents’ final COVID-19 vaccine decisions. As more vaccine evidence becomes available and attempts are made to vaccinate children under 5, parents’ attitudes may alter. Due to the study’s design response rate estimation was impossible. The study’s online survey, which confined participation to those with internet access, may have led to selection bias and monotonous responses from participants who were just trying to get through the survey, resulting in inaccurate and flawed responses. We used convenient sampling method to recruit participants; however, various social media platforms and public visiting places were used to display study questionnaire to avoid selection bias. As such we did not conduct any test to calculate the effect size. Although the English questionnaire was translated to Arabic by professional translator, its psychometric validity was not measured. Finally, because the study’s scope was restricted to Riyadh, its findings cannot be generalized to the rest of the region, nearby provinces, or Saudi Arabia.

## 5. Conclusions

High parental hesitancy to vaccinate their children against COVID-19 was identified, with the novelty of the vaccine and the absence of adequate data regarding its safety serving as the primary concerns. It was found that parents working in healthcare sector, who have heard about anti-vaccine campaign, and who have completed mandatory childhood vaccination were less hesitant towards COVID-19 vaccination. The Saudi health ministry was the primary source of information regarding the COVID-19 vaccination. The future safety and efficacy data should be carefully communicated to parents and healthcare personnel participating in vaccination programs. More than half of the parents were willing to vaccinate their children to prevent them from COVID-19 disease occurrence, yet few parents assumed it was a compulsion from government. Finally, the study’s findings indicate low vaccine acceptance among parents, and hence the healthcare system should develop strategies and interventions to increase the COVID-19 vaccination coverage among children.

## Figures and Tables

**Table 1:** Demographic details of the study participants.

Demographic Information- n = 528			
Characteristics	Category	Number (n)	Percentage (%)
**Parent Age**	25 years and below	41	7.8
	26 to 40 years	358	67.8
	41 years and above	129	24.4
**Relationship with Child**	Father	118	22.3
	Mother	410	77.7
**Nationality**	Saudi	508	96.2
	Non-Saudi	20	3.8
**Marital Status**	Married	483	91.5
	Divorced or separated	45	8.5
**Parents Education**	High school or less	99	18.8
	College degree	373	70.6
	Postgraduate or higher	56	10.6
**Family’s Monthly Income**	Less than SAR 5000	28	5.3
	SAR 5000 to 10,000	116	22.0
	more than SAR 10,000	245	46.4
	Prefer not to answer	139	26.3
**Are you a Healthcare professional?**	Yes	81	15.3
	No	447	84.7
**Job Sector**	Government	178	33.7
	Private	115	21.8
	Self-Employed	39	7.4
	Unemployed	16	3.0
	Retired	27	5.1
	Housewife	146	27.7
	Student	7	1.3
**Number of Children**	One	61	11.6
	Two	109	20.6
	Three	123	23.3
	Four	113	21.4
	Five and above	122	23.1
**Children of 5–11 years age**	Yes	467	88.4
	No	61	11.6
**Gender of children**	Only boys	115	21.8
	Only girls	114	21.6
	Both boys and girls	299	56.6
**Children with chronic diseases?**	Yes	113	21.4
	No	415	78.6

**Table 2:** Association between demographic variables with parents’ willingness to vaccinate their children against COVID-19.

		Willingness to Vaccinate Children Against COVID-19					
Variables	Sub-Group	Not Willing to Vaccinate (%)	Undecided (%)	Delay for a Year and Above (%)	Delay for Few Months (%)	Yes, as soon as Possible (%)	*p* Value
**Parent Age**	25 years and below	11 (26.8)	10 (24.4)	7 (17.1)	3 (7.3)	10 (24.4)	0.206
	26 to 40 years	151 (42.2)	86 (24)	51 (14.2)	22 (6.1)	48 (13.4)	
	41 years and above	41 (31.8)	29 (22.5)	21 (16.3)	10 (7.8)	28 (21.7)	
**Relationship with Child**	Father	32 (27.1)	27 (22.9)	22 (18.6)	15 (12.7)	22 (18.6)	0.004 *
	Mother	171 (41.7)	98 (23.9)	57 (13.9)	20 (4.9)	64 (15.6)	
**Nationality**	Saudi	196 (38.6)	120 (23.6)	75 (14.8)	34 (6.7)	83 (16.3)	0.972
	Non-Saudi	7 (35)	5 (25)	4 (20)	1 (5)	3 (15)	
**Marital Status**	Married	187 (38.7)	117 (24.2)	72 (14.9)	31 (6.4)	76 (15.7)	0.696
	Divorced or separated	16 (35.6)	8 (17.8)	7 (15.6)	4 (8.9)	10 (22.2)	
**Parents Education**	High school or less	32 (32.3)	20 (20.2)	20 (20.2)	11 (11.1)	16 (16.2)	0.198
	College degree	149 (39.9)	94 (25.2)	52 (13.9)	22 (5.9)	56 (15)	
	Postgraduate or higher	22 (39.3)	11 (19.6)	7 (12.5)	2 (3.6)	14 (25)	
**Family’s Monthly Income**	Less than SAR 5000	10 (35.7)	7 (25)	5 (17.9)	1 (3.6)	5 (17.9)	0.023 *
	SAR 5000 to 10,000	35 (30.2)	36 (31)	21 (18.1)	12 (10.3)	12 (10.3)	
	more than SAR 10,000	89 (36.3)	57 (23.3)	32 (13.1)	17 (6.9)	50 (20.4)	
	Prefer not to answer	69 (49.6)	25 (18)	21 (15.1)	5 (3.6)	19 (13.7)	
**Are you a Healthcare professional?**	Yes	16 (19.8)	18 (22.2)	16 (19.8)	8 (9.9)	23 (28.4)	<0.001 *
	No	187 (41.8)	107 (23.9)	63 (14.1)	27 (6)	63 (14.1)	
**Job Sector**	Government	62 (34.8)	37 (20.8)	31 (17.4)	10 (5.6)	38 (21.3)	0.040 *
	Private	32 (27.8)	29 (25.2)	17 (14.8)	14 (12.2)	23 (20)	
	Self-Employed	15 (38.5)	9 (23.1)	6 (15.4)	4 (10.3)	5 (12.8)	
	Unemployed	9 (56.3)	2 (12.5)	2 (12.5)	1 (6.3)	2 (12.5)	
	Retired	11 (40.7)	9 (33.3)	2 (7.4)	Zero	5 (18.5)	
	Housewife	70 (47.9)	38 (26)	20 (13.7)	6 (4.1)	12 (8.2)	
	Student	4 (57.1)	1 (14.3)	1 (14.3)	Zero	1 (14.3)	
**Number of Children**	One	29 (47.5)	10 (16.4)	7 (11.5)	2 (3.3)	13 (21.3)	0.330
	Two	37 (33.9)	27 (24.8)	18 (16.5)	12 (11)	15 (13.8)	
	Three	41 (33.3)	30 (24.4)	19 (15.4)	8 (6.5)	25 (20.3)	
	Four	40 (35.4)	28 (24.8)	22 (19.5)	6 (5.3)	17 (15)	
	Five and above	56 (45.9)	30 (24.6)	13 (10.7)	7 (5.7)	16 (13.1)	
**Children of 5–11 years age**	Yes	185 (39.6)	110 (23.6)	69 (14.8)	31 (6.6)	72 (15.4)	0.488
	No	18 (29.5)	15 (24.6)	10 (16.4)	4 (6.6)	14 (23)	
**Gender of children**	Only boys	51 (44.3)	20 (17.4)	17 (14.8)	10 (8.7)	17 (14.8)	0.401
	Only girls	36 (31.6)	32 (28.1)	15 (13.2)	9 (7.9)	22 (19.3)	
	Both boys and girls	116 (38.8)	73 (24.4)	47 (15.7)	16 (5.4)	47 (15.7)	
**Children with chronic diseases?**	Yes	35 (31)	22 (19.5)	20 (17.7)	14 (12.4)	22 (19.5)	0.018 *
	No	168 (40.5)	103 (24.8)	59 (14.2)	21 (5.1)	64 (15.4)	

* *p* < 0.05.

**Table 3:** Parental behavior, vaccination related to COVID-19 and immunization history of their children.

Parental Behavior, Immunization and Precautionary Measures Related to COVID-19			
	Participant’s Response	Frequency	Percentage (%)
**Have you ever heard about or seen the campaign against COVID- 19 vaccination (anti-vaccination movements)?**	Yes	328	62.1
	No	200	37.9
**Describe your family’s commitment to the precautionary measures against the COVID-19?**	No commitment	55	10.4
	Little commitment	191	36.2
	Somewhat commitment	150	28.4
	Much committed	118	22.3
	Great deal of commitment	14	2.7
**Did anyone within your direct family get infected with COVID-19?**	Yes	399	75.6
	No	129	24.4
**How severe were the symptoms of the infected person(s)?**	Very mild/asymptomatic	46	11.5
	Mild	96	24.1
	Moderate	156	39.1
	Severe	93	23.3
	Death	8	2.0
**As a parent did you take the COVID-19 vaccine?**	Yes	488	92.4
	No	40	7.6
**Did you get any adverse reaction after vaccination?**	Mild	167	31.6
	Moderate	286	54.2
	Severe	35	6.6
**Child/Children’s immunization history**			
**Have you vaccinated your child with mandatory childhood vaccines?**	Yes	411	77.8
	No	117	22.2
**Adverse reactions after vaccination in child?**	Mild	176	42.8
	Moderate	194	47.2
	Severe	41	10.0

**Table 4:** Drivers of parental COVID-19 vaccine hesitancy for their children, drivers of parental willingness to vaccinate their children against COVID-19, or factors influencing parents’ willingness to vaccinate their children against COVID-19.

Driver	Responses		Intention to Vaccinate a Child Against COVID-19				
		Not Willing to Vaccinate (%)	Undecided (%)	Delay for a Year and above (%)	Delay for Few Months (%)	Yes as Soon as Possible (%)	*p* Value
**Have you ever heard about or seen the campaign against COVID-19 vaccination (anti-vaccination movements)?**	Yes	118 (36)	70 (21.3)	49 (14.9)	24 (7.3)	67 (20.4)	0.011 *
	No	85 (42.5)	55 (27.5)	30 (15)	11 (5.5)	19 (9.5)	
**As a parent did you take the COVID-19 vaccine?**	Yes	186 (38.1)	115 (23.6)	69 (14.1)	33 (6.8)	85 (17.4)	0.079
	No	17 (42.5)	10 (25)	10 (25)	2 (5.0)	1 (2.5)	
**Describe your family’s commitment to the precautionary measures against the COVID-19**	No commitment	19 (34.5)	16 (29.1)	10 (18.2)	3 (5.5)	7 (12.7)	0.407
	Little commitment	68 (35.6)	48 (25.1)	32 (16.8)	14 (7.3)	29 (15.2)	
	Somewhat commitment	53 (35.3)	37 (24.7)	23 (15.3)	7 (4.7)	30 (20)	
	Much committed	57 (48.3)	20 (16.9)	12 (10.2)	9 (7.6)	20 (16.9)	
	Great deal of commitment	6 (42.9)	4 (28.6)	2 (14.3)	2 (14.3)	0 (0.0%)	
**Did anyone within your direct family get infected with COVID-19?**	Yes	151 (37.8)	89 (22.3)	61 (15.3)	30 (7.5)	68 (17.0)	0.395
	No	52 (40.3)	36 (27.9)	18 (14.0)	5 (3.9%)	18 (14.0)	
**Previous vaccination in child: Have you vaccinated your child with mandatory childhood vaccines?**	Yes	140 (34.1)	97 (23.6)	65 (15.8)	29 (7.1)	80 (19.5)	<0.001 *
	No	63 (53.8)	28 (23.9)	14 (12.0)	6 (5.1)	6 (5.1)	

* *p* < 0.05.

**Table 5:** Identifying the variables associated with parental intention to vaccinate their children.

Independent Variables	Variable Coefficient (B)	*p*-Value	OR (95% CI) Unadjusted ^a^	OR (95% CI) Adjusted ^b^
***Parental Vaccine Hesitancy against COVID-19 (YES)***				
**Parents relationship with child**				
**Father**	0.105	0.736	0.807 (0.473–1.378)	1.047 (0.572–1.918)
**Mother**	-	-	1.00	1.00
**Family’s monthly income**				
**SAR <5000**	−0.555	0.349	0.728 (0.247–2.148)	0.574 (0.180–1.832)
**SAR 5000–10,000**	0.419	0.316	1.372 (0.636–2.961)	1.520 (0.670–3.448)
**SAR >10,000**	−0.211	0.509	0.618 (0.347–1.097)	0.810 (0.434–1.513)
**Prefer not to disclose**	-	-	1.00	1.00
**Parent Working as Healthcare Professional**				
**Yes**	−0.697	0.040 *	0.414 (0.238–0.718)	0.498 (0.264–0.942)
**No**	-	-	1.00	1.00
**Job Sector**				
**Government**	0.497	0.666	0.614 (0.072–5.25)	1.644 (0.172–15.74)
**Private**	0.413	0.721	0.667 (0.076–5.814)	1.51 (0.157–14.58)
**Self employed**	0.703	0.568	1.133 (0.112–11.48)	2.02 (0.181–22.6)
**Unemployed**	1.10	0.423	1.167 (0.088–15.45)	3.0 (0.204–44.18)
**Retired**	0.569	0.647	0.733 (0.071–7.53)	1.77 (0.155–20.12)
**Housewife**	1.34	0.248	1.861 (0.207–16.76)	3.82 (0.394–37.07)
**Student**	-	-	1.00	1.00
**Children suffering from chronic disease**				
**Yes**	−0.226	0.454	0.754 (0.441–1.29)	0.798 (0.442–1.44)
**No**	-	-	1.00	1.00
**Anti-vaccine campaign**				
**Yes**	−0.993	0.001 *	0.409 (0.237–0.704)	0.371 (0.210–0.654)
**No**	-	-	1.00	1.00
**Mandatory childhood Immunization**				
**Yes**	−1.614	<0.001 *	0.224 (0.095–0.527)	0.199 (0.081–0.488)
**No**	-	-	1.00	1.00
**Vaccine Hesitancy score (VHS)**				
**Hesitant: VHS score <3 points**	0.458	0.083	0.594 (0.366–0.964)	0.633 (0.377–1.062)
**Not Hesitant: VHS score ≥3 points**	-	-	1.00	1.00

^a^ The ORs for unadjusted analyses are from the univariate logistic regression analysis. ^b^ The ORs for adjusted analyses are from the multivariable logistic regression analysis including significant factors from the univariate analysis. * *p* < 0.05.

## Data Availability

The datasets generated and/or analyzed during the current study are not publicly available due to ethical restrictions but are available from the corresponding author on reasonable request.
